# Critical appraisal of a brief 5 item version of the Neck Disability Index

**DOI:** 10.1186/1477-7525-12-34

**Published:** 2014-03-10

**Authors:** Charles P Gabel, Antonio I Cuesta-Vargas, Markus Melloh, Jason Osborne

**Affiliations:** 1 Faculty of Science, Health and Education, Centre for Healthy Activities, Sport and Exercise, University of the Sunshine Coast, Sippy Downs Dve, Sippy Downs, Sunshine Coast Qld 4556, Australia; 2 Universidad de Malaga, Andalucia Tech, Facultad de Ciencias de la Salud, Departamento de Psiquiatria y Fisioterapia, Instituto de Biomedicina de Malaga (IBIMA), Grupo de Clinimetria (AE-14) Av/ Arquitecto Peñalosa s/n (Teatinos Campus Expansion), 29009 Malaga, Spain; 3 Queensland University Technology, School Clinical Sciences, Kelvin Grove, QLD 4059 Brisbane, Australia; 4 Educational and Counseling Psychology, University of Louisville, 2323 S Brook St, Louisville, KY 40208, USA; 5 Centre for Medical Research (CMR), Faculty of Medicine, Dentistry and Health Sciences, The University of Western Australia, 35 Stirling Highway, Crawley WA 6009 Perth, Australia

## Dear editor

We congratulate Walton and MacDermid [1] for their insightful proposal and brief 5-item Neck Disability Index (NDI-5). Their methodology addresses recognized NDI [2] validity problems including the dual factor structure [3], face and content validity from poor WHO-ICF compliance [4] and ordinal scale values due to descriptive response options [5].

The proposed NDI-5 overcomes these by providing a single factor structure ensuring all items can summate to a single score, WHO-ICF compliance through the conceptual reductive process, and interval scale values achieved from Rasch analysis.

The NDI problems highlighted focus concerns on the results from publications where the primary outcome is a single functional patient-reported outcome (PRO) measure, such as the NDI. Such large-scale, prospective trials can influence policy, funding, and opinions on the effectiveness of interventions, therapies, and professions [6]. When such study results prefer one approach over another, based on a single PRO with validity concerns, the PRO’s clinimetric characteristics must be considered and why concurrent PROs were not used in such studies.

The stated NDI problems also raise questions for the NDI-5 items (personal care, concentration, work, driving, and recreation). The employed reductive conceptual retention methodology is well recognized [7]; however, the retained NDI-5 items differ notably from those identified by other authors using this methodology [8,9]. Walton and MacDermid acknowledged this: “…item removal was liberal and other authorship groups may [arrive] at a different scale…”. Our recent study used Confirmatory Factor Analysis (CFA) [9] and determined two functional categories, retaining ‘Physical’ (personal care, lifting, work, driving, sleep, and recreation) and deleting ‘Mental’ (pain, headache, concentration, and reading). Consequently, four items agree, but we retained ‘sleep’ under ICF codes b134 and d410 and ‘lifting’ under d430, while ‘concentration’ was removed. Furthermore, the NDI-5 ‘driving’ item was “…modified…to read ‘driving/riding in a vehicle’, in recognition of…the population who do not drive” and reduced from six to five options. The authors stated they “…don’t believe this change…adversely affect[s] the properties of the scale”; however, “…the results…were achieved through use of the original wording.”

These incongruities raise three potential new issues for the proposed NDI-5:

1) Face and content validity as the NDI-5 retains ‘concentration’ and four ‘Physical Function’ items, with ‘driving’ modified in both language and score options.

2) Clinimetric characteristics are unknown as the NDI-5 is yet to be prospectively tested and analysed.

3) Final score proportionality is affected as changing one item from six to five responses weights the other items disproportionately. Selectively disadvantaging one of five scale items is particularly problematic as all summed items should have identical response options.

Four further considerations implied within this study must be highlighted.

First, generalization to large populations is difficult and as Walton and Macdermid note “Confidence…will be increased when replicated in an independent cohort.” This homogenous physiotherapy mechanical neck pain population has only two-thirds with work status, cross-gender differential item functioning further reduces potential samples, and only 50 participants were examined for ‘longitudinal validity’. Though reasonable, the samples are proportionally small, a common problem in many outcome clinimetric studies.

Second, in the Rasch analyses, criterion for exclusion was not stated. Thus, there may have been items eliminated that technically were significant but practically not unreasonable. Additionally, the authors identify overall goodness of fit (Chi Square = 89.1) as being relatively poor when the Chi-square:*df* ratio was well within reasonable range (CHI/DF ≈ 0.28).

Third, to minimize bias the reduction process should ideally have had objective experts as highlighted by differences with the CFA study selected items [9].

Finally, full validation of the NDI-5 should include criterion-related validity and a direct comparison with the NDI-10 to determine whether the new proposed version is superior to the existing or other proposed reduced-versions.

Consequently the “…ripe directions for future research…” fall into three categories:

I) Concurrent PRO use as Walton and MacDermid concluded through use of “…patient-centred…[and]…symptom-specific scales…to allow separation of important constructs while maximizing sensitivity to change.” These concurrent PROs should consider the entire spinal kinetic chain (e.g. Spine Functional Index [10], Functional Rating Index [11]) or use software-based PROs from mobile computers and smart phones that employ computerized adaptive testing (FOTO) [12] or more progressively, decision support systems [13] integrating predictive screening and outcome use (AdviseRehab) [14] with the capacity to incorporate “…symptom-specific items”.

II) Conversion tables as provided with the NDI-5 [1] and NDI-7 [5].

III) Recommendations on future tools with sound clinimetrics: fulfilling all necessary criteria [15,16] including both psychometrics [17] and practicality [18], independently researched and sufficiently progressive as to be incorporated and used within available and future technology.

The NDI has served researchers, clinicians, and patients well. The discussed limitations are determined from evolutionary processes considering clinimetrics and evidence determination. We must not lose what the NDI has contributed but retain the benefits and move forward. The essential components of evidence [19] must remain and be supported by validity and critical insight from PROs that are adaptable and progressive in our technologically connected age.

Yours Sincerely

Charles Philip Gabel, Antonio Cuesta-Vargas, Markus Melloh and Jason Osborne

## Competing interests

The authors declare that they have no competing interests.

## Authors’ contributions

All the authors have made contributions to conception and drafting of this letter. All the authors have given final approval of the version to be published.
